# Visual Advantage in Deaf Adults Linked to Retinal Changes

**DOI:** 10.1371/journal.pone.0020417

**Published:** 2011-06-01

**Authors:** Charlotte Codina, Olivier Pascalis, Chris Mody, Peter Toomey, Jill Rose, Laura Gummer, David Buckley

**Affiliations:** 1 Academic Unit of Ophthalmology and Orthoptics, University of Sheffield, Sheffield, United Kingdom; 2 Laboratoire de Psychologie et NeuroCognition, Université Pierre Mendes France, Grenoble, France; 3 Department of Ophthalmic Imaging, Sheffield Teaching Hospitals Trust, Sheffield, United Kingdom; Washington University, United States of America

## Abstract

The altered sensory experience of profound early onset deafness provokes sometimes large scale neural reorganisations. In particular, auditory-visual cross-modal plasticity occurs, wherein redundant auditory cortex becomes recruited to vision. However, the effect of human deafness on neural structures involved in visual processing prior to the visual cortex has never been investigated, either in humans or animals. We investigated neural changes at the retina and optic nerve head in profoundly deaf (N = 14) and hearing (N = 15) adults using Optical Coherence Tomography (OCT), an in-vivo light interference method of quantifying retinal micro-structure. We compared retinal changes with behavioural results from the same deaf and hearing adults, measuring sensitivity in the peripheral visual field using Goldmann perimetry. Deaf adults had significantly larger neural rim areas, within the optic nerve head in comparison to hearing controls suggesting greater retinal ganglion cell number. Deaf adults also demonstrated significantly larger visual field areas (indicating greater peripheral sensitivity) than controls. Furthermore, neural rim area was significantly correlated with visual field area in both deaf and hearing adults. Deaf adults also showed a significantly different pattern of retinal nerve fibre layer (RNFL) distribution compared to controls. Significant correlations between the depth of the RNFL at the inferior-nasal peripapillary retina and the corresponding far temporal and superior temporal visual field areas (sensitivity) were found. Our results show that cross-modal plasticity after early onset deafness may not be limited to the sensory cortices, noting specific retinal adaptations in early onset deaf adults which are significantly correlated with peripheral vision sensitivity.

## Introduction

A lifetime of sensory deprivation, as experienced by profoundly and congenitally deaf individuals can induce sometimes large-scale neural reorganisations within sensory cortices [1]. Such plasticity influences the remaining senses, with visual sensitivity in the congenitally deaf selectively enhanced as a result [2]. Although neural reorganisations concerning the sensory cortices of early onset deaf adults are widely reported, the effect of human deafness on neural structure involved in visual processing prior to the visual cortex has not so far been investigated either in humans or animals. We investigated such neural plasticity at the retina and optic nerve in deaf and hearing humans using the non-invasive technique of Ocular Coherence Tomography (OCT) to image and quantify retinal microstructure and test whether retinal changes relate to differences in peripheral vision sensitivity.

Specific changes have been reported in the visual and auditory cortices of early onset, profoundly deaf adults. The auditory association cortex of deaf humans shows activation to watching sign language [3] and the right auditory cortex including primary auditory cortex (A1) shows activation to visual stimuli [1], [4], [5]. Significantly different scalp distributions of event-related brain potentials (ERPs) to peripheral visual motion and colour stimuli have been reported in deaf adults, with occipital cortex responses from deaf participants 5–6 times higher than in controls [6]. In deafened mice auditory cortical neurons have been shown to respond to somatosensory and visual stimulation and the size of A1 is significantly increased [7]. Visual responses in otherwise classically defined auditory regions of the brain have also been demonstrated in ferrets deafened from birth [8]. There is some debate as to whether a visual cortical hypertrophy may also occur in the deaf. In humans however, one fMRI study [4] found only auditory-visual cortical plasticity and not visual hypertrophy in response to vision. A recent study [9] investigated cross-modal plasticity in deaf and hearing cats and compared results with behavioural changes and found that peripheral vision sensitivity was significantly increased in the deaf cats, and furthermore that enhanced abilities could be traced to neural correlates in the deaf auditory cortex and not the visual cortex.

These cortical changes in response to vision appear to influence deaf visual behaviour, promoting selective visual enhancements in deaf adults, specifically resourcing peripheral vision. A range of peripheral visual tasks has been tested wherein deaf perform better in aspects such as enhanced motion processing, reorienting visual attention, and enhanced detection of fine object or luminance changes in the visual periphery in the deaf [6], [10]–[15]. Deaf adults have also been found to detect a fine kinetic light stimulus at further peripheral locations than hearing controls [16], [17].

We suggest the retina as an additional site for plasticity in the deaf because it is far from fully developed at birth, requiring an unimpeded optical system to achieve normal development [18], [19] and because peripheral vision in hearing individuals appears to receive input from auditory stimuli at cortical level, suggesting convergence of the auditory and visual stimuli in cortical structures [20]. Retinal ganglion cell (RGC) types present in human retina at birth migrate substantially across the retina until 4 years old, with the fovea having an immature appearance and all retinal cell types present at the fovea prior to migration [21]–[24]. Additionally, refinement of retinal circuitry continues for a substantial time period into childhood and requires activity modulation to achieve normal status^25^. Neural retina also remodels in response to retinal degenerations, initially by subtle changes to neural structure and later by large scale reorganisations including neuronal and glial migration, elaboration of new neurites and synapses, and neuronal cell death and rewiring of retinal circuits [25]. Remodelling is found in multiple species in which RGCs initially extend dendrites through multiple sublaminae and later modify their arbors to achieve laminar specificity by an activity dependent process, whereas others are confined to destination laminae from the outset [26]–[30]. Thus the retina of congenitally deaf infants may undergo specialised arborisation and adapted competition from neighbouring ganglion cells in the ultimate number and destination of RGCs.

The sensory experience of a deaf child clearly varies from that of a hearing child, but what (if any) effect this has on the retina is not known. The field of peripheral vision to which typically developing children are able to attend, increases throughout childhood, becoming adult-like in the far periphery to dim stimuli at around 11–12 years [31]. Development of peripheral vision in deaf children differs significantly from hearing controls, with young deaf children (aged 5–8 years) slower to detect and report fewer peripheral targets, but the difference between deaf and hearing children reduces, with rapid compensation such that by 13 years of age deaf adolescents were faster than hearing controls [31]. These developmental peripheral visual changes in deaf children may be influenced by altered sensory developmental changes.

RGC number is significantly correlated with neural rim area [32], [33]. Therefore neural rim area provides an accurate non-invasive measure of retinal neural structure which directly correlates to RGC number and these measures can also be directly compared with peripheral visual sensitivity [34]. In measuring aspects of the optic nerve head and surrounding peripapillary retina, we test whether retinal structure may differ between deaf and hearing individuals; and whether retinal structural change may relate to the observed increase in peripheral vision sensitivity in deaf adults.

## Results and Discussion

There was no significant difference between the visual acuities of the deaf and hearing participants as tested with the Bailey-Lovie eye chart [35] (p = 0.67). All further analyses were conducted on the right eye only to avoid over estimations of statistical significance, as no significant differences were found on any results between right and left eyes.

### Optic nerve head analyses

**Figure 1:**
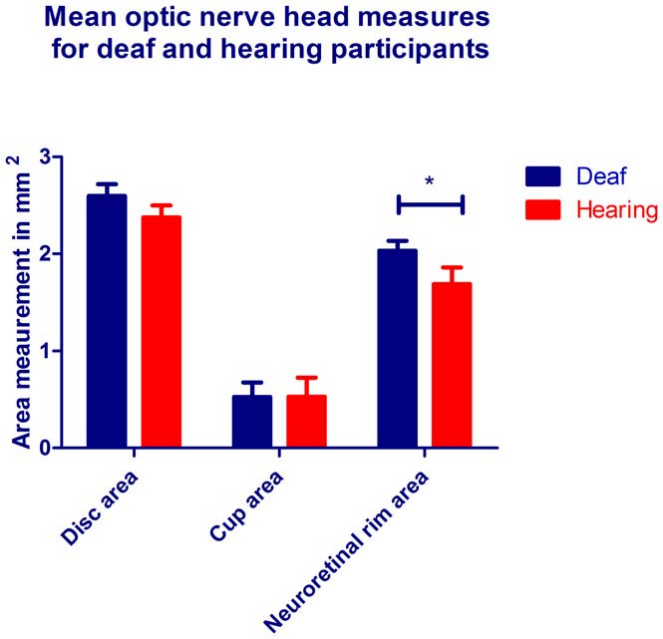
Mean areas of optic nerve head measures for deaf and hearing participants. Bars indicate areas in mm^2^ for the deaf (blue) and hearing (red) participants. Measurements were taken from the six radial optic nerve head scans described and error bars denote the standard error of the mean (SEM). Due to the non-normative statistical behaviour of area data, raw data measurements were root squared for statistical analysis.

We conducted optic nerve head analyses using ocular coherence tomography (OCT) on all deaf and hearing participants to address whether the increased neural substrate to vision robustly demonstrated at cortical level, may extend to increased neural substrate within the optic nerve. The overall outcome of these scans can be seen in Figure 1 where mean areas of the optic cup, optic disc and neural rim are shown in mm^2^. As can be seen the measures were generally larger in the deaf group: disc area (2.60vs 2.37 mm^2^), neural rim area (2.03vs1.69 mm^2^) and optic cup area (0.53vs0.53 mm^2^). Due to the non-normative behaviour of area data, data were root squared before analyses were conducted. Three separate t-tests were used to test for significant difference between deaf and hearing groups. Neural rim area was significantly thicker in the deaf than in the hearing participants (t = 2.221, p = 0.034), but differences between the optic cup (t = 1.704, p = 0.098) or optic disc (t = 2.00, p = 0.054) areas in deaf vs. controls were not significant.

We are confident these OCT results may represent retinal plasticity in the deaf, because the measurements of neuroretinal rim area were taken by six repeated and carefully centred OCT scans of the optic nerve head with excellent participant fixation after pupil dilation in every case. In addition, OCT shows excellent correlation with histological examinations of the human retina and neuroretinal rim area in particular, is directly related to the number of RGC axons within the optic nerve [14], [32], [33]. Therefore significantly increased neural rim area in deaf adults is consistent with previous reports of increased neural substrate in the deaf [1], [3], [5], [9] and furthermore suggests that increased neural resourcing to vision may additionally be present in the optic nerve. In support of this, one study [36] reported high inter-individual correlation for the sizes of the optic tract, LGN and visual cortex within the neural anatomy of 15 human individuals. Thus, a large V1 primary visual cortex was associated with a large LGN and large optic tract, yet a two-three fold variation in component size for all structures was found between individuals. In a further study by the same groups of authors [37], a threefold variation in visual discrimination ability amongst healthy emmetropic adults was reported with the suggestion that the coordinated variations in visual component size could potentially be responsible for substantial sensory variations in visual ability. If increased visual cortical mapping in deaf individuals is associated with an increase in the optic tract, one may expect to find an increase in the size of the optic disc area as well. Indeed the optic disc area was larger in the deaf participants, though just under statistical significance and a higher number of participants may yet reveal this finding. A three-fold inter-individual variation in photoreceptor density amongst healthy human eyes has been reported and suggested as the cause for size variations in cortical visual representations [38]. Thus, density of retinal structure may relate to cortical visual area representation, which in turn is associated with coordinated increases to more anterior neural visual pathway structure, for example the optic nerve and retina.

**Figure 2:**
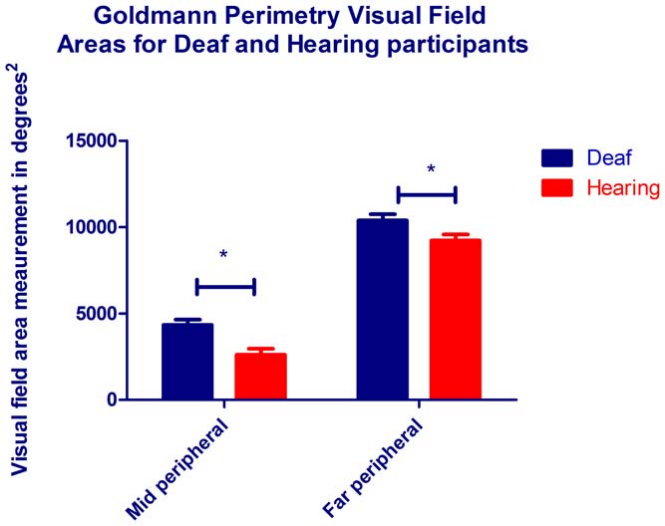
Mean visual field areas for deaf and hearing participants. Bars indicate areas in degrees^2^ for the mid peripheral (2Ie Goldmann stimulus of luminance 20 cds/m^2^, area 0.25 mm^2^) and far peripheral (4Ie Goldmann stimulus of luminance 328 cds/m^2^, area 0.25 mm^2^) visual fields for the deaf (blue) and hearing (red) participants. Error bars denote SEM and raw data were root squared prior to statistical analysis due to the non-normative behaviour of area data.

It was important to test whether the increase in peripheral visual sensitivity previously documented in deaf adults was also present in the adults from this study. Of the deaf and hearing participants who participated in OCT, 8 deaf (6M, 2F, mean age 33.1 yrs) and 10 hearing (8M, 2F, mean age 30) participants underwent assessment of their visual field sensitivity using Goldmann perimetry (see experimental procedures for details). Figure 2 clearly shows that the mean visual field areas were larger for the deaf participants for both the mid-peripheral (4327.68°^2^ vs 2607.81.68°^2^) and far-peripheral fields (10384.01°^2^ vs 9209.1°^2^). A two factor ANOVA was conducted on the root squared raw data where the first factor was visual field (mid peripheral or far peripheral) and the second factor was group (deaf or hearing). As expected, the effect of visual field was significant (F_1,64_ = 226.7, p<0.001), and deaf showed significantly larger visual fields (F_1,64_ = 14.64, p<0.0001). The difference between deaf and hearing visual fields was significant for the mid-peripheral visual field (t = 3.464, p = 0.015) and for the far peripheral visual field (t = 2.346, p = 0.041).

Consistent with previous reports [16], [17] deaf adults showed significantly increased visual field areas for both the mid- and far- peripheral fields on Goldmann perimetry compared to hearing controls. The increase in peripheral vision sensitivity is also consistent with several other reports which detail such aspects as enhanced motion processing, and enhanced detection of fine object or luminance changes in the visual periphery in the deaf [6], [10]–[13]. The visual field advantage in deaf adults has not previously been linked to any change at the retina or optic nerve. It has however, been linked to auditory cortex activation during peripheral viewing [9], with the same study finding that when the auditory cortex was deactivated by a cooling procedure, the deaf peripheral visual advantage was no longer present, therefore discovering a neural correlate for peripheral vision in the deaf. We now suggest the optic nerve as an additional correlate to this advantage. To test the significance of the optic nerve structure on the visual function performance we asked the question “Is neural rim area related to peripheral vision performance?”

### Visual field area and neuroretinal rim area correlations

**Figure 3:**
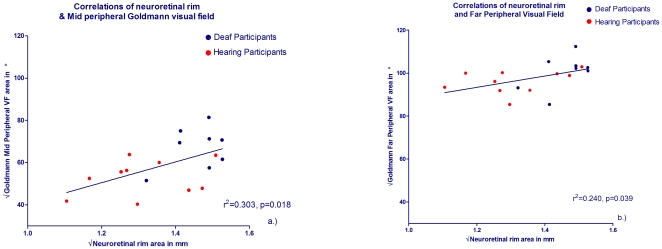
Optic nerve neural rim and visual field area correlations for deaf and hearing participants. Square root of the neural rim area is shown on the x axes for deaf participants (blue symbols) and hearing participants (red symbols) for a.) mid-peripheral visual field (2Ie Goldmann stimulus, area 0.25 mm^2^ , luminance 20 cd/m^2^) and b.) far-peripheral field (4Ie stimulus, area 0.25 mm^2^, luminance 328 cd/m^2^).

Using root squared raw data we plotted individuals' neural rim area (x axes) against visual field area (y axes) for both the mid-peripheral visual field and far-peripheral visual field areas and applied linear regressions to both. As can be seen in Figure 3, the deaf (blue plots) had both larger neural rim areas and larger visual field areas than the hearing participants (red plots). The two variables were positively correlated with significant correlation for neural rim area and mid-peripheral Goldmann visual field area (r^2^ = 0.303, p = 0.018) (Figure 3a) and significant for the far-peripheral Goldmann peripheral field (r^2^ = 0.240, p = 0.039) (Figure 3b). There were no significant correlations using the other optic nerve head measures of cup or disc area with the mid- or far-peripheral visual field (largest r^2^ = 0.083).

In Figure 3 we document for the first time a significant linear correlation between the neural rim area and visual field area amongst adult deaf and hearing individuals, thus showing a clear relationship between increased neural substrate and increased peripheral vision sensitivity. The correlation coefficients for the mid-peripheral field and for the far-periphery whilst significant, cannot account for all the variance within these data, suggesting that additional factors may contribute to the observed visual field increase in the deaf. Indeed, age of participant and change to the distribution of attentional load may represent such factors. The relationship between optic nerve structure and visual field function has been well documented previously, with visual field testing at specific peripheral locations showing good correlation with the relative location of those test points on peripheral retina and the corresponding bundle numbers of RNFL at the optic nerve head both in healthy and glaucomatous eyes [39], [40]. However, the relationship between structure and function has only been reported for standard automated perimetry in the mid-periphery using the Humphrey field analyser, and has never been reported using kinetic visual field perimetry in the further visual periphery as used in this study. Here, we find that the well evidenced peripheral vision enhancement in the deaf is related to changes in the retinal structure in both deaf and hearing participants.

### RNFL analyses

**Figure 4:**
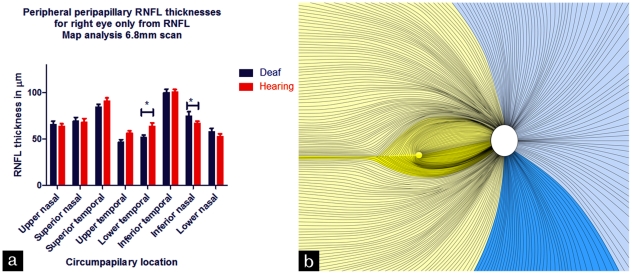
Circumpapillary RNFL measurements for deaf and hearing participants and corresponding retinal locations. Figure 4a shows the eight locations at which RNFL measurements were taken on the y axis for deaf participants (blue) and hearing participants (red) for the right eye only. Error bars denote SEM. 4b illustrates these circumpapillary locations by highlighting in bold the two retinal locations at which significant differences were found between deaf and hearing participants. Pale yellow overlay denotes temporal hemi-retina relating to the nasal binocular visual field in which areas hearing showed thicker RNFL than deaf; pale blue denotes nasal hemi-retina relating to the temporal monocular visual field in which areas deaf showed thicker RNFL than hearing. The black lines indicate the right eye retinal nerve axonal pathways from the ganglion cells to the optic disc (OD; indicated by the white oval). This image has been adapted and shaded from Hogan MJ, Alvarado JA, Weddell JE (1971) Histology of the Human Eye An Atlas and Textbook, W.B. Saunders Company p536.

We further assessed any neural differences at the retina in the deaf by analysing the depth of the retinal nerve fibre layer (RNFL) across 4 cardinal and 4 inter-cardinal areas circumferential to the optic nerve head. Figure 4a shows the 8 areas around the optic nerve head at which RNFL thickness was measured, showing a difference in the RNFL distribution between deaf and hearing participants and Figure 4b shows a schematic representation of the retina, with yellow overlay denoting regions where hearing participants showed thicker RNFL and blue overlay denoting the regions where deaf had thicker RNFL. A two factor mixed measures analysis of variance (ANOVA) was conducted to analyse the data where the first factor was group (deaf or hearing) and the second factor was retinal area (the 8 peripapillary areas at which RNFL thickness was measured) on the 6.8 mm scan (see Experimental Procedures). A separate ANOVA was conducted for data from the 2.9 mm scan, but significant differences were found only at 6.8 mm, where RCGs are further towards destination retinal locations (see Figure 4b). For the 6.8 mm scan there was no significant effect of hearing status on overall RNFL thickness (p = 0.334). However, significant interaction was found between hearing status and retinal location (F_7,210_ = 2.282, p = 0.021). Post-hoc t-tests corrected for multiple analyses by Bonferroni adjustment were conducted between deaf and hearing participants at each retinal location. In Figure 4b the bold yellow illustrates the peripapillary region at which RNFL was significantly thicker for hearing participants (t = 2.48, p = 0.04). This region is immediately temporal to the optic nerve head and contains the papillomacular bundle. The bold blue colouring marks the inferior nasal peripapillary region wherein RNFL was significantly thicker for deaf participants (t = 2.713, p = 0.041). Interestingly, this region serves the far monocular temporal visual field.

Thus we found a significant interaction between deafness and retinal location on RNFL analyses. The significant decrease to RNFL thickness in deaf adults occurred in temporal retina in a region containing the papillomacular bundle which supplies the fovea. Interestingly, there was no significant difference between the visual acuities of deaf and hearing and several studies have failed to find a difference in central visual abilities between deaf and hearing individuals [1], [41]–[43]. However, the increased density of RGCs at the fovea and macula in healthy human retina suggests the presence of a RGC reserve or redundancy such that structural damage to the optic nerve secondary to glaucoma may precede any damage to visual function [39]. Therefore there may be some reduction of temporal peripapillary RNFL possible before any loss in function becomes detectable. An exception to this occurs in studies which have carefully manipulated visual spatial attention in deaf and hearing individuals and compared abilities to detect subtle visual differences centrally and at various near peripheral locations. In these studies deaf adults have shown an increased attentional ability in the visual periphery, whereas hearing adults performed significantly better than deaf when the attentional load was manipulated to involve a central vision change [13], [44], [45]. Profoundly deaf adults have been found to be more proficient at a task which requires ignoring foveally presented stimuli [46]. One study [47] found a neural correlate to this attentional shift, reporting that deaf individuals had decreased activity of cortical area MT-MST (medial temporal/medial superior temporal) in response to central vision, and increased activation of MT-MST to peripheral motion processing, compared to hearing controls. Here we find a retinal correlate to these documented changes in visual spatial processing, showing that the RNFL is directed preferentially towards monocular and most peripheral visual field areas and reduced towards areas of central vision such as in the papillomacular bundle. We suggest that absence of auditory input may drive not only MT-MST adaptations but retinal adaptations in order to capture more visual peripheral information.

**Figure 5:**
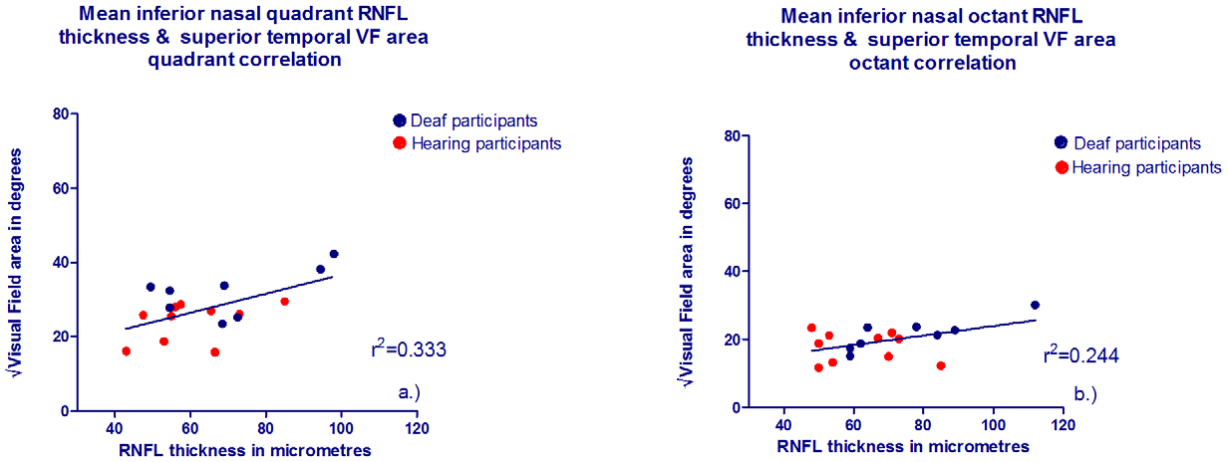
RNFL thickness and corresponding visual field quadrant/octant area correlations for deaf and hearing participants. The x axis shows RNFL thickness in µm for deaf (blue symbols) and hearing (red symbols) for the root squared of a.) Inferior nasal quadrant and corresponding superior temporal mid-peripheral visual field quadrant area and b.) Inferior nasal octant and corresponding superior temporal mid- peripheral visual field octant area in degrees.

The inferior nasal retinal location where deaf adults had increased RNFL compared to hearing corresponds to the superior temporal and far temporal monocular visual field [34], [48]–[53]. Therefore inferior nasal circumpapillary RNFL thickness and superior-temporal visual field area were tested for possible correlation. Figure 5a shows a scatterplot of individuals' inferior nasal quadrant RNFL thickness against superior temporal quadrant (root squared) mid-peripheral visual field area and these two measures showed significant correlation (r^2^ = 0.333, p = 0.012). The mean inferior octant RNFL thickness also showed significant correlation with the mid peripheral superior temporal visual field octant area (Figure 5b, r^2^ = 0.244, p = 0.037). Interestingly, no other correlations were significant for other circumpapillary regions where RNFL was not significantly different between deaf and hearing when tested for relationship with corresponding visual field areas (highest r^2^ = 0.197).

There is therefore further evidence that the structural change in RNFL at retinal locations at which deaf show increased RNFL is related to a sensitivity increase in the corresponding visual field. Retinal adaptation in the deaf which directs the neural layer preferentially towards the monocular temporal visual field, as opposed to the binocular nasal field may be mediating a specific advantage to further peripheral vision in deaf individuals. This increase in retinal neural substrate to far peripheral vision is consistent with the previously noted deaf advantage both in this and previous studies. It has also been noted that when deaf individuals monitor peripheral rather than central visual stimuli, neural activity is particularly stimulated [6], [9], [54]. The superior temporal octant and quadrant of the mid-periphery and far-periphery were not significantly different between deaf and hearing adults by t-tests in that region of the visual field only. However, this could be due to the relatively small number of participants from the OCT study who also completed the Goldmann perimetry visual field assessment. Whilst the visual field has been mapped successfully for the immediate 60° of view [50], the very peripheral retina has not been mapped to the optic nerve head and our results suggest that the relationship continues into the further peripheral field and may additionally be recognised with kinetic perimetry. Individuals may deviate from the average map of visual field regions and corresponding RNFL sectors defined by [50], but relatively little is known about this [39]. Indeed deaf individuals may represent such a deviation from normal RNFL patterns.

## Discussion

Our data suggest a relationship between retinal structure in terms of optic nerve neural rim area, RNFL organisation, and functional sensitivity in peripheral vision. Notably the neural rim area of the deaf emmetropic adults was significantly increased compared to controls and significantly correlated with sensitivity in both the mid-peripheral and far-peripheral visual field. RCG number, determined by RNFL thickness appeared preferentially distributed to nasal hemi-retina in the deaf, which relates to the monocular temporal field and was significantly correlated with peripheral visual sensitivity in the corresponding visual field quadrant or octant (see figure 5). This relationship between retinal structure and visual specific enhancements in deaf individuals has not, to the best of our knowledge been previously shown.

This study replicated previous reports of increased visual field size in profoundly deaf adults [16], [17]. The visual field increase documented in the deaf is in close agreement with specific peripheral vision enhancements previously reported in deaf adults [2], [9]. In deaf, but not hearing participants visual stimuli have caused activation in the auditory cortex and increased activation in motion selective area MT/MST, however it remained undetermined if the compensatory effects observed in visual function were enhanced by increased resourcing to peripheral retina and the optic nerve. Our results suggest that both the retina and optic nerve adapt to allow further peripheral information to be captured prior to the increased visual processing at cortical level which has been previously evidenced.

Rather than neural resourcing being uniformly increased across the whole retina in deaf persons, our results suggest that the RNFL may reorganise to preferentially distribute RGCs to the far monocular temporal visual field and even subtly reduce the RGC resource to central vision. This observed reorganisation is not dissimilar to other retinal reorganisations in response to a very different form of sensory deprivation in amblyopia [55] and is consistent with the migration of different cell types observed across the retina until 45 months of age [56]. It has been argued that visual functions that are most likely to reorganise after early onset deafness are those which would under normal development would benefit from the convergence of both auditory and visual stimuli [2]. Peripheral vision, used to monitor the surrounding environment for change or hazards would normally benefit from simultaneous auditory and visual information, therefore in the absence of hearing, the peripheral vision magnocellular pathway must compensate for both senses. Indeed peripheral vision in hearing individuals appears to receive input from auditory stimuli at cortical level suggesting convergence of the auditory and visual stimuli in cortical structures [20].The temporal visual field has previously been seen to increase in deaf adults whereas the nasal hemi-field has not shown such a large increase [17]. When one monitors the visual environment, it is the far temporal visual field which would be likely to alert the viewer of a change or hazard in the extreme periphery, and in this visual field area we observed an increase which correlated with the thickness of the RNFL in corresponding nasal retina.

Because this is the first study relating retinal changes in the deaf to visual compensations, we thought it worthy to consider another sensory deprivation condition that leads to change in the structure of retina, LGN and cortex as well as reduction to central vision function. Amblyopia results in a reduction to central visual acuity and contrast sensitivity function and in line with this the optic nerve neural rim area in amblyopic eyes in comparison to non-amblyopic patients is significantly reduced, resulting in increased retinal receptor areas [57]. One study [55] reported that amblyopic eyes had slightly greater foveal minimum thickness than the normal fellow eye, yet interestingly this difference in thickness reduced when amblyopia was successfully treated and visual acuity improved. Poorer responses of LGN cells from amblyopic eyes have been reported [58] over those cells driven by the normal eye together with the suggestion that reorganisation of the LGN pathways or even the retina could be responsible for such change. Hubel [59] described that geniculate layers receiving afferents from a deprived eye after 3–6 days of visual deprivation appeared thinner and smaller with atrophied cell bodies compared to those from the fellow eye. Cortical cells responding to an amblyopic eye show reduced spatial resolution and contrast sensitivity [19]. Thus in amblyopia, visual development is detracted and changes are found within all visual pathway structure with retinal receptor area increased as a result, whereas for deafness in which vision is selectively enhanced, we also have observed developmental changes in more anterior visual structures, specifically at the optic nerve and retina.

Evidence suggests that substantial loss of neurones and synapses characterises normal neural development [60], and that neuronal activity specifies selective elimination and maintenance of cortical connections. RGCs comprising RNFL are present at birth and migrate towards specific retinal destinations in the post-natal months with maturation of these cells and the post-receptoral pathways continuing over the first 45 post-natal months [56]. One explanation for retinal plasticity is a different pattern of migration for these cell types in deaf infancy in response to the altered sensory experience of profound deafness; leading to increased resourcing to temporal peripheral vision and therefore reduced retinal receptor areas in far peripheral vision and greater peripheral sensitivity. We think that we have observed the end result of this plasticity process in deaf adults. All except one deaf subject in this study had been diagnosed with profound deafness by age 18 months; a time period in which retinal development remains incomplete. The fate of the retinal ganglion cell types and final retinal locations is thought to be directed by retinal progenitors, yet no intrinsic factors have been identified specifying retinal ganglion cell fate within the eye [61]. Only four of the deaf group were known to be genetically deaf, although several of the group had unknown cause for deafness which could include an unidentified genetic precursor. A genetic adaptation which not only causes deafness but affects change at retinal ganglion cell level is a possibility but cannot hold for all deaf participants in this study as there were no RNFL differences between the genetically deaf group and the remainder when analysed separately. One participant from the deaf group became deaf at age 4 years and therefore the data were retested excluding this participant, however this made no difference to the significance levels of the results. This stage of becoming deaf was beyond the stage classically defined as ‘early onset’, yet is still within the scope of maturations of the retina to take place. This participant's results show similarity to the other deaf participants and therefore this raises interest for when these retinal changes may be occurring. The visual field advantage in deaf individuals is later to arise than expected, first identified at 11 years old by one study [62] and 13 years old by another [31]. Therefore if hearing loss occurred after 4 years old, although current theory would suggest that retinal maturation is complete, continued development of peripheral vision suggests that a peripheral visual advantage may be possible even in those whose onset of deafness is beyond 4 years old. The mechanism for this continued development is yet to be investigated, but may include post-receptoral and LGN and cortical development as well as improved attention to peripheral space. A longitudinal case study, performing OCT on deaf and hearing children in association with visual field testing is suggested in light of these findings.

In summary, our results suggest an extension of the well established neural adaptation to deafness found at the cortex, to be considered at the optic nerve and retina. We suggest a causal relationship between increased neural substrate in the form of optic nerve neural rim area increase, RNFL preferential distribution towards temporal peripheral vision and the peripheral vision sensitivity enhancement shown by deaf adults in this and other studies.

## Materials and Methods

Optical coherence tomography (OCT) is an objective, in-vivo light interference method of quantifying retinal micro-structure and was used in this study to measure retinal nerve fibre layer (RNFL) thickness circumferential to the optic nerve and to evaluate the optic nerve head in terms of optic disc area, optic cup area, and the area of the neuroretinal rim.

This research was carried out in accordance with the declaration of Helsinki and approved by the North Sheffield NHS Ethics Centre of Research and Ethics Campaign (COREC) UK, who wrote “This project has been reviewed by the Research Department and authorised by the Medical Director on behalf of Sheffield Teaching Hospitals (STH) NHS to begin”. Informed written consent was obtained from all participants prior to entry into the study. The deaf group (N = 14, 10 male, 4 females, mean age 30.4 years) all had profound degree binaural deafness diagnosed before 4 years of age that was of sensorineural cause, not attributed to systemic disorders known to affect the eye with no participant having cochlear implants. The hearing group (N = 18, 14 males, 4 females, mean age 30.0 years) were recruited from colleagues with none having hearing deficits. Both groups were emmetropic with excellent visual acuities in either eye of at least 0.100 LogMAR (6/7.5 Snellens equivalent). All participants underwent baseline testing of visual acuity, pupillary reactions and fundus examinations. No participant had any significant ophthalmic history nor any signs or history of glaucoma. Further details of the patients can be found in Table 1. It is worth noting that 2 participants became deaf as a result if in-uterine Rubella and one as a result of meningitis, which both could have caused a visually associated impairment. However on careful ophthalmic and Orthoptic investigations no defects were found.

**Table 1:** Further information regarding deaf participants.

*Gender*	*Age*	*1st Language*	*Cause of Deafness*	*Age became deaf*	*Parents deaf?*
F	31	BSL	Genetic	Birth	Y
M	42	English	Maternal rubella	Birth	N
M	22	BSL	Unknown	Birth	N
M	21	English	Unknown	4 years	N
M	39	English	Maternal rubella	Birth	N
M	19	English	Unknown	Birth	N
M	32	English	Genetic	Birth	Mother deaf
M	35	English	Unknown	Birth	N
M	20	English	Unknown	Birth	N
F	27	English	Genetic	Birth	Mother deaf
M	29	English	Unknown	Birth	N
F	40	BSL	Genetic	Birth	N
F	25	English	Meningitis	2 years	N
F	26	English	Unknown	Birth	N

Table 1 Details the cause and onset of deafness, as well as native language for the 14 deaf participants in our study.

Stratus OCT (Carl Zeiss Meditec, Dublin California, USA) scans were taken at 2.9 mm and 6.8 mm diameters across the optic nerve head after pupil dilation with Tropicamide 1% in both right and left eyes. The scans were taken 6 times and a mean of the measurements of each scan was used. No differences were found between the data from the two eyes, taken at the 8 locations and on optic nerve head analyses, consistent with [63]. Therefore only data from the right eye was used to avoid over estimations of the statistical significance of the results. Hood and Kardon [39] have documented the effects of misaligning the circumpapillary circular scan either horizontally or vertically which can shift the peaks of the RNFL scans temporally or nasally, or even decrease the amplitude of the peaks of the scan. For this reason, care was taken when performing the OCT scans and the results are not consistent with any of the patterns of misalignment reported [39]. It was not possible to obtain additional normative ocular coherence tomography results from the manufacturers for comparison.

Of the 32 participants who participated in OCT measurements, 18 participants (8 deaf, 10 hearing) underwent Goldmann perimetry for either eye which measured the extent of the mid-peripheral and far-peripheral visual fields. The mid-peripheral visual field was measured to the 2Ie target which of stimulus area 0.25 mm^2^ luminance 20 cds/m^2^ candelas and the far peripheral field was measured to the 4Ie target which is stimulus area 0.25 mm^2^, luminance 328 cds/m^2^. The participant maintains central fixation to a central target which is ensured by the examiner via a telescope. The light stimulus is then introduced in the far periphery of the Goldmann perimeter and travels slowly at 3–5 msec^−1^ towards the central target. The participant presses a buzzer when the peripheral stimulus is first seen in the visual periphery and the position at which the participant first reported the stimulus is recorded. Thus each of the two kinetic stimuli were moved slowly towards the participant's point of central fixation every 15° around the visual field in random order. The visual field areas for the mid-peripheral and far peripheral visual fields were then calculated by the areas of each triangle, comprised by the two adjacent meridian locations at which the light stimuli were first seen. For further details on the methodology of the visual field assessment, please see [17].
